# Morphological Transformation of Myeloma Cells into Multilobated Plasma Cell Nuclei within 7 Days in a Case of Secondary Plasma Cell Leukemia That Finally Transformed as Anaplastic Myeloma

**DOI:** 10.1155/2017/5758368

**Published:** 2017-12-21

**Authors:** Akihito Fujimi, Yasuhiro Nagamachi, Naofumi Yamauchi, Yuji Kanisawa

**Affiliations:** ^1^Department of Hematology, Sapporo Kiyota Hospital, Sapporo, Japan; ^2^Department of Hematology and Oncology, Oji General Hospital, Tomakomai, Japan

## Abstract

A 48-year-old man was diagnosed with multiple myeloma (IgG-k) and was treated with high-dose dexamethasone as an induction therapy followed by thalidomide-based regimens. Approximately 22 months after the initial diagnosis, the patient developed secondary plasma cell leukemia (PCL) with a white blood cell (WBC) count of 20.2 × 10^9^/L including 79.5% plasma cells. A G-banding chromosomal analysis in the bone marrow showed an t(11;14) abnormality of up to 5%, which was not detected at initial diagnosis. We immediately started bortezomib and dexamethasone therapy, but in just 7 days, the WBC count elevated to 48.5 × 10^9^/L, and approximately 95% of them were medium-sized atypical lymphoid cells with multilobated nuclei. Although we subsequently initiated alternative regimens, the patient's condition deteriorated, and he died 4 months after developing PCL. Approximately 2 months before his death, the diameter of myeloma cells in the bone marrow enlarged by approximately twofold, and pleomorphic nuclei were present, indicating an anaplastic myeloma transformation. Concurrently, a 100% increase of the t(11;14) clone frequency was observed in the G-banding-analyzed bone marrow cells. Morphological transformation of myeloma cells into multilobated plasma cell nuclei can be considered as the starting point of the sequential process leading to anaplastic myeloma.

## 1. Introduction

Anaplastic myeloma is an extremely rare, but distinct, subtype of myeloma with poorly differentiated, pleomorphic, and significantly enlarged plasma cells [1–3]. The prognosis of anaplastic myeloma is extremely poor, with a mean survival of less than 3.5 months [2]. Another morphological variation of multiple myeloma described as multiple myeloma with cleaved, convoluted, or multilobated plasma cell nuclei has also been reported previously [4–13], having been frequently associated with plasma cell leukemia (PCL) [4–8] and with poor prognosis as well [6–10]. To date, the correlation between anaplastic myeloma and other morphological types of multiple myeloma has not been reported. Here, we present a case of secondary PCL, which morphologically transformed into multilobated plasma cell nuclei just 7 days after bortezomib and dexamethasone administration, and finally, transformed into anaplastic myeloma that simultaneously occurred with an expanded t(11;14) chromosomal abnormality.

## 2. Case Presentation

**Figure 1:**
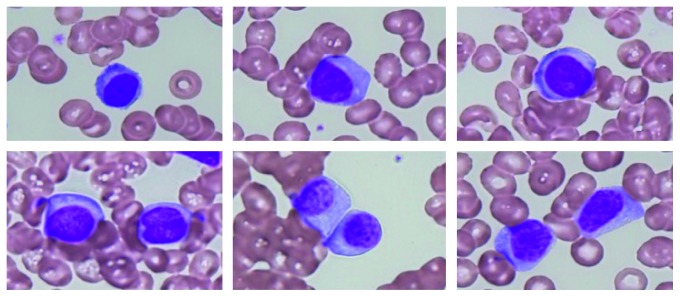
Morphological changes in myeloma cells immediately after the development of plasma cell leukemia. The WBC count was 20.2 × 10^9^/L, and 79.5% of them were composed of typical myeloma cells (1,000x, May–Grünwald-Giemsa staining).

**Figure 2:**
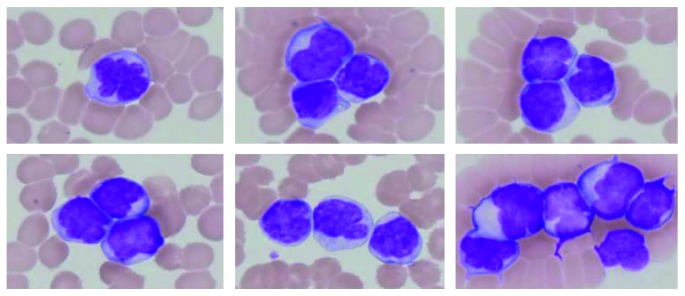
Morphological changes in myeloma cells seven days after initiating bortezomib and dexamethasone. The WBC count was elevated to 48.5 × 10^9^/L, and 95% of them were composed of medium-sized atypical lymphoid cells with multilobated nuclei (1,000x, May–Grünwald-Giemsa staining).

**Figure 3:**
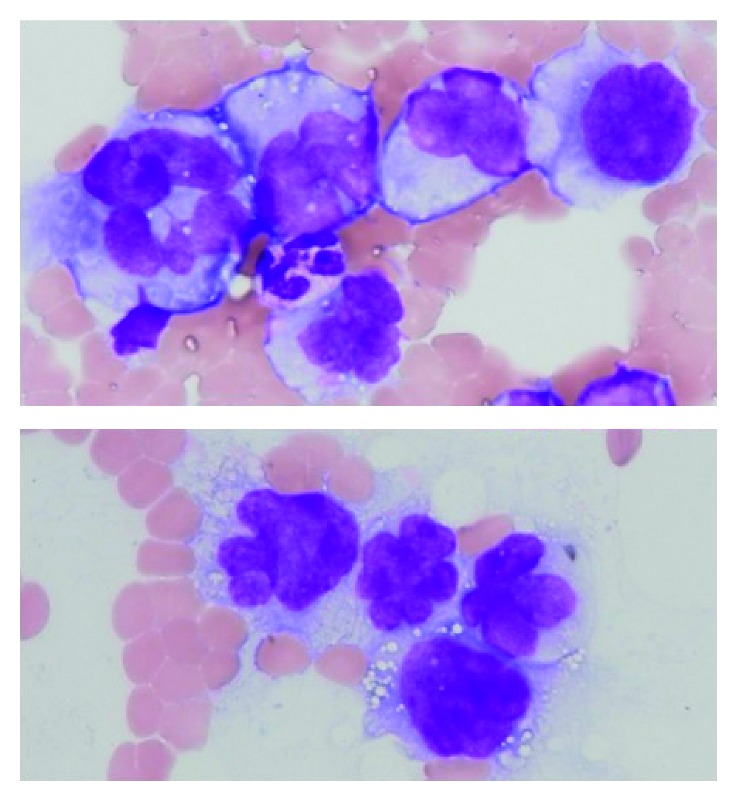
Morphological changes in myeloma cells two months after the development of secondary plasma cell leukemia. The diameter of the myeloma cells in the bone marrow significantly enlarged by approximately twofold, and pleomorphic nuclei and vacuoles in the cytoplasm were observed, indicating a transformation into anaplastic myeloma (1,000x, May–Grünwald-Giemsa staining).

A 48-year-old man was diagnosed with multiple myeloma (IgG-k, Durie-Salmon IIIB, ISS III) in February 2008 and was treated with one course of high-dose dexamethasone as an induction therapy followed by TD (thalidomide and dexamethasone) therapy for the succeeding 4 months. G-banding chromosomal analysis at the initial diagnosis showed normal diploid cells. High-dose chemotherapy with autologous peripheral blood stem cell transplantation was not performed as stem cell mobilization failed despite the use of high-dose cyclophosphamide and granulocyte colony-stimulating factor. As a result, thalidomide 50 mg was continued for 8 months and then switched to MPT (melphalan, prednisolone, and thalidomide) therapy after disease progression. In December 2009, after seven courses of MPT therapy, the patient developed secondary PCL (Figure 1). The following laboratory results were obtained: white blood cell (WBC) count, 20.2 × 10^9^/L (plasma cells, 79.5%); hemoglobin level, 11.4 g/dL; platelet count, 2.9 × 10^4^/L; and IgG, 3,770 mg/dL in the peripheral blood. Chromosomal analysis by G-banding showed t(11;14) abnormality of up to 5% in the bone marrow: 45, -X, -Y, add(1)(q32), -11, der(14)?t(11;14)(q13;q32), +2mar [1]/ 46, XY [19]. Extramedullary plasmacytoma was not observed on whole body computed tomography. About 2 weeks earlier, no evidence of PCL was noted in the peripheral blood: WBC count, 3.2 × 10^9^/L (plasma cells, 0.0%); hemoglobin level, 12.3 g/dL; and platelet count, 9.6 × 10^4^/L. BD (bortezomib 1.3 mg/m^2^, days 1, 4, 8, 11 and dexamethasone 20 mg, days 1, 2, 4, 5, 8, 9, 11, 12 of 21) therapy was immediately initiated; however, only 7 days after, laboratory data revealed an increased WBC count (48.5 × 10^9^/L) with approximately 95% of them being medium-sized atypical lymphoid cells with multilobated nuclei (Figure 2). During this period, no decrease in WBC count and no evidence of tumor lysis syndrome were observed. To elucidate the cell origin, a flow cytometry of the peripheral blood was performed, which showed the same phenotypes as the previously identified myeloma cells from the bone marrow. Namely, these cells expressed CD38, CD138, CD45, MPC-1, and cytoplasmic Igκ and did not express CD3, CD4, CD8, CD10, CD19, CD20, CD49e, CD56, and cytoplasmic Igλ. Although several regimens were given, such as 3 courses of VAD (vincristine, Adriamycin, and dexamethasone) and 4 subsequent courses of MMCP (ranimustine, melphalan, cyclophosphamide, and prednisolone), the patient's condition continuously deteriorated further, and he eventually died 4 months after developing PCL. Approximately 2 months before he died, the diameter of myeloma cells in the bone marrow increased twofold, and pleomorphic nuclei and vacuoles in the cytoplasm developed (Figure 3), indicating a transformation into anaplastic myeloma. Furthermore, a 100% increase of the t(11;14) clone frequency was observed in the bone marrow cells. The result of chromosomal analysis by G-banding was as follows: 45, -Y, del(X)(q?), ins(1;?)(q21;?), t(11;14)(q13;q32) [16]/ 45, sl, add(3)(q27), del(6)(q?) [2]/ 46, sl, +1, -ins(1;?), der(5)t(1;5)(q12;q35), +12 [1]/ 45, sl, +1, -ins(1;?), der(21)t(1;21)(q12;q22)[1]. Concurrently, the myeloma cells in the peripheral blood remained to be medium-sized atypical lymphoid cells with multilobated nuclei. Extramedullary plasmacytoma was not observed throughout the clinical course.

## 3. Discussion

Anaplastic myeloma can be diagnosed during the initial visit or may develop as the disease progresses [1–3], a characteristic similar to other low-grade lymphoid malignancies [14, 15]. Meanwhile, previous studies of myeloma cells with cleaved, convoluted, or multilobated nuclei reported that these cells have been present at the initial diagnosis in all cases [4–13]. In the present case, the development of secondary PCL, morphological transformation of myeloma cells into multilobated nuclei, and anaplastic transformation subsequently emerged in a short period of time, which resulted in the patient's death only 2 months after the anaplastic transformation. Therefore, the morphological transformation of myeloma cells into multilobated nuclei could be considered as the starting point of the sequential process leading to anaplastic transformation.

Interestingly, the morphological transformation of myeloma cells emerged in just 7 days in the current case. Although the possibility that a small clone with multilobated nuclei expanded rapidly in a short period cannot be ruled out, we speculate that each myeloma cell simultaneously underwent a morphological transformation because the myeloma cell count increased only by 2.8-fold within 7 days versus the development of morphological transformation of myeloma cells in almost all the myeloma cells. About 2 weeks before the development of secondary PCL, no myeloma cells were evident in the peripheral blood, that is, the secondary PCL developed rapidly within 2 weeks. Thus, we suspect that these myeloma cells might have originated from a same clone in the bone marrow, and each of the cells might have aggressive characteristics resistant to BD therapy, with a predisposition for morphological transformation. Although the transformation happened just after the initiation of BD therapy, it might have occurred owing to the disease progression, and a possibility of the transformation being induced by BD therapy is low.

As the prognosis of anaplastic myeloma is poor, novel therapies, such as proteasome inhibitor or immunomodulatory drugs, are anticipated. Agrawal et al. reported a case of anaplastic myeloma with end-organ dysfunction effectively treated with bortezomib-based chemotherapy [16], whereas Ammannagari et al. reported two cases of anaplastic myeloma, of which one was resistant to bortezomib-based chemotherapy, and the other was resistant to bortezomib and lenalidomide combined chemotherapy [17]. In this case, bortezomib-based chemotherapy was ineffective for secondary PCL, and morphological transformation also occurred just after the treatment. At that time, lenalidomide was yet to be approved in Japan.

The treatment resistance and aggressive behavior of anaplastic myeloma can be associated with the high frequency of unfavorable cytogenetic abnormalities. Bahmanyar et al. reported that the prevalence of 1q21 amplification, 17p deletions, and t(4;14) in anaplastic myeloma was significantly higher than that in nonanaplastic myeloma [18]. They also reported that t(11;14) chromosomal abnormality was found in 18% cases of anaplastic myeloma, although it was not statistically significant compared with that of the nonanaplastic myeloma cases (13%) [18]. Some t(11;14)-associated anaplastic myeloma cases have also been reported. Maslovsky et al. reported a fulminant anaplastic myeloma case with complex karyotype, including t(11;14) [19], and Ammannagari et al. also reported an anaplastic myeloma case with t(11;14) refractory to bortezomib and lenalidomide combined chemotherapy [17]. In the present case, t(11;14) chromosomal abnormality that was not detected during initial diagnosis emerged concurrently with the occurrence of secondary PCL and progressed rapidly along with the anaplastic transformation. In the era of novel therapies, t(11;14) chromosomal abnormality is not considered as an unfavorable karyotype of multiple myeloma [20]; however, it was detected in a substantial proportion of primary or secondary PCL cases, which is an aggressive subset of multiple myeloma [21]. Thus, t(11;14) chromosomal abnormality is suspected to be associated with the morphological transformation of myeloma cells into anaplastic type.

This is the first reported case of myeloma cells being morphologically transformed into multilobated nuclei during the clinical course and simultaneously followed by anaplastic myeloma transformation. Further investigation regarding the morphological transformation of myeloma cells and the possible correlation with t(11;14) chromosomal abnormality is required.
